# RON Receptor Tyrosine Kinase Regulates Epithelial Mesenchymal Transition and the Expression of Pro-Fibrotic Markers via Src/Smad Signaling in HK-2 and NRK49F Cells

**DOI:** 10.3390/ijms20215489

**Published:** 2019-11-04

**Authors:** Jung Sun Park, Hoon-In Choi, Dong-Hyun Kim, Chang Seong Kim, Eun Hui Bae, Seong Kwon Ma, Soo Wan Kim

**Affiliations:** Department of Internal Medicine, Chonnam National University Medical School, 42 Jebongro, Gwangju 61469, Korea; gene-pjs@hanmail.net (J.S.P.); hoonin_c@hanmail.net (H.-I.C.); dhkim450@gmail.com (D.-H.K.); laminion@hanmail.net (C.S.K.); baedak76@gmail.com (E.H.B.); drmsk@hanmail.net (S.K.M.)

**Keywords:** recepteur d’origine nantais, epithelial-mesenchymal transition, pro-fibrotic marker, Src pathway, kidney

## Abstract

Receptor tyrosine kinases (RTKs) play important roles in the pathogenic processes of kidney fibrosis. However, the pathophysiological roles of recepteur d’origine nantais (RON), one of the receptor tyrosine kinases, have not yet been defined. We investigated whether the activation or sequence-specific small interfering RNA (siRNA) suppression of RON could regulate epithelial mesenchymal transition (EMT) and the expression of pro-fibrotic markers, and its underlying molecular mechanisms. Stable cell lines and transient transfection for RON and the transfected cells of siRNA for RON were developed to investigate the molecular mechanisms in human kidney proximal tubular epithelial (HK-2) and interstitial fibroblasts (NRK49F) cells. RON overexpression induced EMT and increased expression of fibrosis-related proteins such as N-cadherin, vimentin, transforming growth factor-β (TGFβ), αSMA, and fibronectin in HK-2 and NRK49F cells. RON overexpression increased various RTKs and the phosphorylation of Src (Y416) and Smad, while inhibition of RON by siRNA attenuated the expression of EMT- and fibrosis-related proteins and decreased RTKs such as insulin-like growth factor receptor (IGFR), fibroblast growth factor receptor 1 (FGFR1), vascular endothelial growth factor receptor (VEGFR), and platelet-derived growth factor receptor (PDGFR), as well as the phosphorylation of Src and Smad pathways. siRNA silencing of Src also attenuated the expression of IGFR, FGFR1, VEGFR, and PDGFR. Inhibition of RON can exert an anti-fibrotic effect by the inhibition of EMT and other RTKs through control of Src and Smad pathways in HK-2 and NRK49F cells.

## 1. Introduction

Chronic kidney disease (CKD) can lead to the development of kidney dysfunction and require costly therapy with dialysis or transplantation, and, furthermore, is associated with high rates of morbidity and mortality [1]. According to the global trends of CKD from 1990 to 2016, the prevalence of CKD is increasing due to population growth and aging. The incidence of CKD is much more pronounced in low- or middle-income countries around the world [2]. Compared to an average cost of treating disease, CKD has a 1.8-fold burden on medical costs due to complications such as diabetes, hypertension, stroke, heart disease, and infection. Therefore, CKD is closely related to aging and chronic diseases, and its socio-economic burden continues to increase [3,4]. In this context, slowing the progression of kidney disease can prevent various complications, save on medical costs, and lead to patients living healthier lives.

Kidney fibrosis is a common pathway for the progression of kidney disease. The most common causes of CKD are diabetes or hypertension, which account for more than two-thirds of CKD [5]. As is well known, diabetes and hypertension lead to inflammation by increasing tumor necrosis factor (TNF)-α, transforming growth factor (TGF)-β, angiotensin II, reactive oxygen species, and hypoxia by induction of nuclear factor-κB (NF-κB) in kidney cells [6,7]. Increased inflammatory reaction occurs in almost all cell types in the kidneys, including fibroblasts, tubular epithelial cells, pericytes, endothelial cells, mesangial cells, and podocytes, as well as the infiltrated cells such as lymphocytes, macrophages, and fibrocytes [8,9]. The accumulation of these cells associated with inflammation may lead to the epithelial mesenchymal transition (EMT) of the tubular epithelial cells [10,11], and the proliferation and transdifferentiation of interstitial fibroblasts to myofibroblasts, which may be the major contributors to kidney fibrosis [12].

Receptor tyrosine kinases (RTKs) regulate a variety of cell physiological processes, including metabolism, growth, differentiation, and survival [13]. However, the abnormal phosphorylation of RTKs increases intracellular signaling, causing cancer, inflammation, and fibrosis in the kidney [14,15]. Important RTKs related to kidney fibrosis are platelet-derived growth factor receptor (PDGFR), vascular endothelial growth factor receptor (VEGFR), epidermal growth factor receptor (EGFR), discoidin domain receptor (DDR), Axl receptor (AXLR), and insulin-like growth factor receptor (IGFR). Currently, among various studies by RTKs inhibition, monoclonal antibodies or tyrosine kinase inhibitors are used to aid in the prevention and treatment of kidney fibrosis. In particular, imatinib, a PDGFR inhibitor, reduced pathologic changes in different models of CKD; fibroblast growth factor receptor (FGFR) inhibitor AG1296 inhibited the proliferation of kidney fibroblasts; and Erlotinib, an EGFR inhibitor, significantly reduced TGF-β mediated fibrogenesis [16,17,18]. However, to overcome the limitations in the treatment of kidney fibrosis by the use of monoclonal antibodies or tyrosine kinase inhibitors, there is a need for studies on signaling and gene therapy mediated by them. Some RTKs are not yet well known with regard to kidney fibrosis. Among others, recepteur d’origine nantais (RON) belongs to the hepatocyte growth factor receptor (HGFR) superfamily [19].

RON, known as macrophage-stimulated 1 receptor, is a 180-kDa protein and its ligand is a macrophage-stimulating protein. Upon ligand binding, RON is dimerized and autophosphorylated, and transduces a variety of signals. RON is involved in multiple signaling cascades that mediate adhesion, cell motility, proliferation, and apoptosis as well as increase the expression of hepatocyte growth factor receptor (HGFR) and various genes through the activation of a variety of downstream signaling pathways, such as Src, RAS/mitogen-activated protein kinase (MAPK), phosphatidyl inositol-3 kinase (PI-3K)/AKT, focal adhesion kinase, and NF-κB [20,21,22]. Recent studies have demonstrated the reduction of kidney fibrosis by inhibition of other RTKs, such as EGFR and VEGFR [23,24,25]. However, it has not yet been determined whether RON can regulate kidney fibrosis, and if so, the underlying molecular mechanisms. Thus, we investigated whether RON regulates epithelial mesenchymal transition (EMT) and the expression of pro-fibrotic markers with various signals related to the etiology of kidney fibrosis.

## 2. Results

### 2.1. Expression of RON Is Associated with Fibrosis in UUO Mouse Model

**Figure 1:**
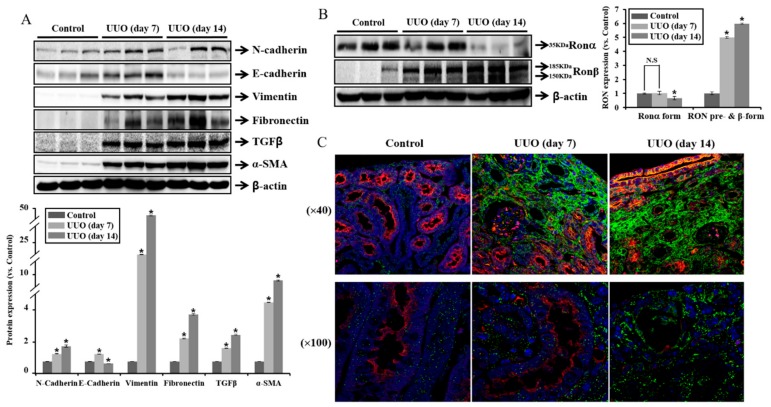
Expression of RON, epithelial mesenchymal transition (EMT), and pro-fibrotic markers in obstructed mouse kidneys. The unilateral ureteral obstruction (UUO) lasted for 7 day and 14 days. (**A**) Protein expression of N-cadherin, E-cadherin, vimentin, fibronectin, TGFβ, and α-SMA were analyzed. (**B**) Protein expression of RONα and RONβ were analyzed. (**C**) RON-receptor tyrosine kinase expression was evaluated by confocal microscopy. The RON expression was observed as a green label. The expression of AQP-1 (red label) was used as a marker of the proximal tubule, and 4′, 6-diamidino-2-phenylindole (DAPI: blue label) was used as a maker of the nuclei. Each column represents the mean ± SEM. * *p* < 0.05, compared with the UUO control group. N.S, statistically not significant.

We studied the expression of receptor tyrosine kinase RON in the kidney of a unilateral ureteral obstruction (UUO) mouse model. Figure 1A shows that the protein expression of N-cadherin and vimentin was increased in the obstructed kidneys after 7 days and 14 days compared with controls, whereas E-cadherin was decreased in the obstructed kidneys after 14 days. The protein expression of fibronectin, TGF-β, and α-SMA was increased at 7 days and 14 days. To determine the changes in RON, we analyzed the expression pattern of RONα and RONβ in the mouse UUO kidneys. RON is synthesized as a 185 kD precursor protein. This proform is then cleaved into the 35 kD extracellular alpha chain and the 150 kD beta chain, which has extracellular, membrane spanning and intracellular domains. The extracellular portion of the beta chain is then disulfide linked to the alpha chain, forming the mature receptor [26].

RONα showed decreased protein level at 14 days of obstructed kidneys as compared to 7 days, and the expression patterns of RONβ and RON precursor forms increased at 14 days (Figure 1B). We further examined the RONβ staining by immunofluorescence. As shown in Figure 1C, green fluorescence of RONβ staining was gradually increased in the obstructed kidneys at 7 days and 14 days compared with controls, and proximal tubular cells showed red fluorescence by aquaporin-1 staining. These finding suggest that RONβ expression was predominantly increased in the peritubular interstitium, which might be associated with the tubulointerstitial fibrosis.

### 2.2. Effect of RON Overexpression in Proximal Tubular HK-2 and Interstitial Fibroblasts NRK49F Cells

**Figure 2:**
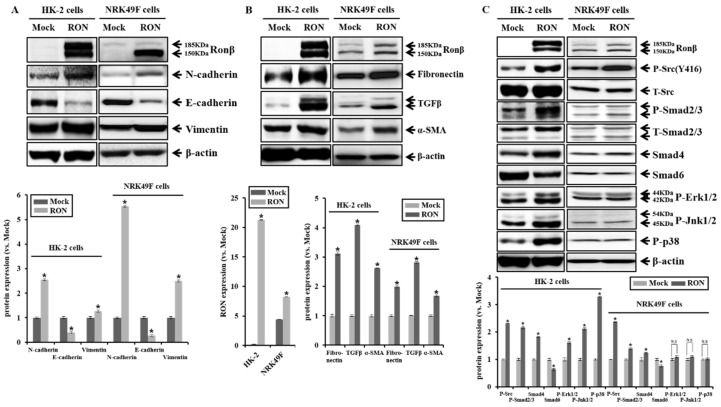
Effects of recepteur d’origine nantais (RON) overexpression on EMT, pro-fibrotic marker, and the Src signaling pathway in HK-2 and NRK49F cells. (**A**) Protein expression of the N-cadherin, E-cadherin, and vimentin by RON overexpression was analyzed. (**B**) Protein expression of fibronectin, TGFβ, and α-SMA by RON overexpression was analyzed. (**C**) Protein expression of phosphorylated Src (Y416) was assessed in RON overexpression and controls. Protein expression of the phosphorylated Smad2/3, Smad4, and Smad6 by RON overexpression was analyzed. Protein expression of phosphorylated ERK (P-ERK), phosphorylated JNK (P-JNK), phosphorylated P38 (P-p38) was assessed in RON overexpression and controls. Each column represents the mean ± SEM. * *p* < 0.05, compared with the Mock. N.S, statistically not significant.

We performed stable transfection of an empty vector (Mock) and a plasmid encoding human RON in the human kidney proximal tubular epithelial (HK-2) cells to examine the physiological effect of RON. The selection of RON stable cell clone was determined via the confirmation of zeocine expression, which was contained in the backbone plasmid *pCDNA4*, and the expression of c-terminal c-Myc tagged RON protein was assessed by anti-c-Myc. RON overexpression in rat kidney interstitial fibroblast (NRK49F) cells was demonstrated by transient transfection. Overexpression of RON increased the active form 150 kD RON β form by four times in NRK49F cells. Overexpression of RON increased the expression of EMT markers such as N-cadherin and vimentin, while E-cadherin was reduced in HK-2 and NRK49F cells. Moreover, the overexpression of RON increased the expression of pro-fibrotic markers, such as fibronectin, TGFβ, and α-SMA in HK-2 and NRK49F cells. (Figure 2A,B). We examined signaling pathways in the RON-overexpressed HK-2 and NRK49F cells. During kidney fibrosis progression, the TGFβ signal has Smad-dependent and Smad-independent signal pathways [27,28]. The Smad-dependent signal pathway exerts its biological effects by activating Smad2/3, which is regulated negatively by an inhibitory Smad6/7. As shown in Figure 2C, Smad2/3 phosphorylation and Smad 4 expression were increased, while Smad6 expression was decreased in RON-overexpressed HK-2 and NRK49F cells. These results indicate that overexpression of RON is associated with TGFβ/Smad-dependent signal transduction. The overexpression of RON also increased the phosphorylation of MAPKs in HK-2 cells.

However, there was no change in MAPK signaling in NRK49F cells. In addition, the overexpression of RON increased the phosphorylation of Src containing the tyrosine kinase catalytic domain. Src can be activated by autophosphorylation at Tyr416, which is induced upon the activation of a wide variety of transmembrane receptor proteins that include the receptor tyrosine kinases, G protein–coupled receptors, integrins, and cytokine receptors [29]. As shown in Figure 2C, the phosphorylation of Src increased in the RON-overexpressed HK-2 and NRK49F cells.

### 2.3. Effects of RON on Other RTKs in Proximal Tubular HK-2 and Interstitial Fibroblast NRK49F Cells

**Figure 3:**
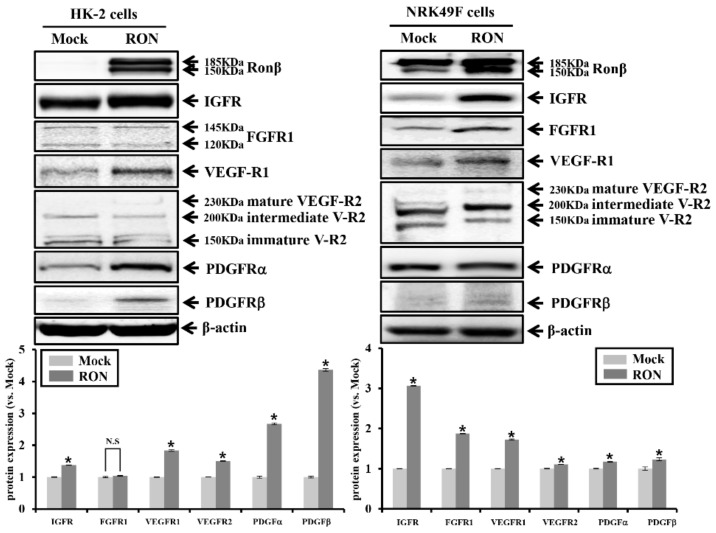
Protein expression of receptor tyrosine kinases (RTKs) by RON overexpression in HK-2 and NRK49F cells. Protein expression of IGFR, FGFR1, VEGFR1, VEGFR2, PDGFRα, and PDGFRβ by RON overexpression was analyzed. Each column represents the mean ± SEM. * *p* < 0.05, compared with the Mock. N.S, statistically not significant. IGFR, insulin-like growth factor receptor; VEGFR, vascular endothelial growth factor receptor; PDGFR, platelet-derived growth factor receptor.

We further examined whether the protein expression of several RTKs was associated with fibrosis in RON-overexpressed HK-2 and NRK49F cells. As shown in Figure 3, the overexpression of RON increased the protein expression of RTKs such as IGFR, FGFR, VEGF-R1, VEGF-R2, PDGFRα, and PDGFRβ in HK-2 and NRK49F cells. We examined RONβ staining by immunofluorescence analysis in the RON-overexpressed HK-2 cells.

**Figure 4:**
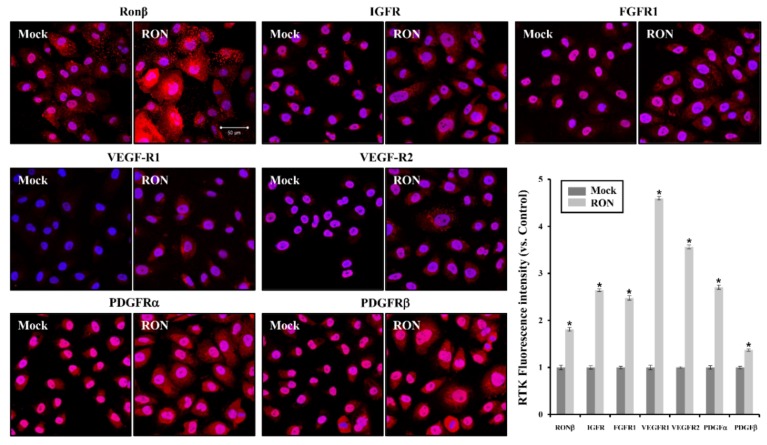
Expression of RTKs by RON overexpression in HK-2 cells. Immunofluorescence of RON, IGFR, FGFR1, VEGFR1, VEGFR2, PDGFRα, and PDGFRβ by RON overexpression was evaluated using fluorescence microscopy. The overexpression of RON (red) significantly increased other RTKs. The nucleus (blue) was stained with DAPI. (magnification, 400×; bar = 50 μm) * *p* < 0.05, compared with the Mock.

As shown in Figure 4, the red fluorescence of various RTKs staining was increased in RON-overexpressed HK-2 cells compared with Mock. These results suggest that RON overexpression is associated with an increase in various RTKs.

### 2.4. Effect of RON siRNA on EMT, Pro-Fibrotic Marker, Src Signaling Pathway in HK-2 and NRK49F Cells

**Figure 5:**
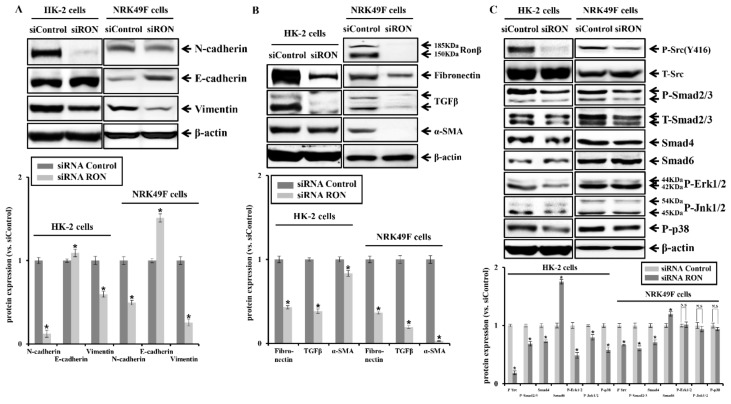
Effects of RON siRNA on EMT, pro-fibrotic marker, and the Src signaling pathway in HK-2 and NRK49F cells. (**A**) Protein expression of N-cadherin, E-cadherin, and vimentin by RON siRNA transfection was analyzed. (**B**) Protein expression of fibronectin, TGFβ, and α-SMA by RON siRNA transfection was analyzed. (**C**) Protein expression of phosphorylated Src (Y416) was assessed in RON siRNA transfection and siControl. Protein expression of phosphorylated Smad2/3, Smad4, and Smad6 by RON siRNA transfection was analyzed. Protein expression of phosphorylated ERK (P-ERK), phosphorylated JNK (P-JNK), and phosphorylated P38 (P-p38) was assessed in RON siRNA transfection and siControl. Each column represents the mean ± SEM. * *p* < 0.05, compared with the siRNA control. N.S, statistically not significant.

RON-specific siRNA treatment decreased the protein expression of EMT markers, such as N-cadherin and vimentin, while the protein expression of E-cadherin was increased in HK-2 and NRK49F cells (Figure 5A). Moreover, RON-specific siRNA treatment reduced the expression of pro-fibrotic markers such as fibronectin, TGFβ, and α-SMA (Figure 5B). Treatment of RON-specific siRNA decreased phosphorylation of Src compared to control siRNA (Figure 5C).

**Figure 6:**
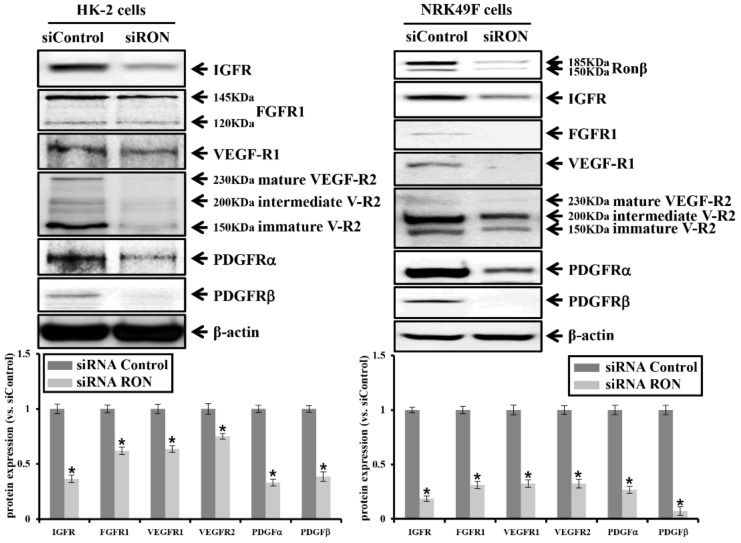
Effects of RON siRNA on RTKs in HK-2 and NRK49F cells. Protein expression of IGFR, FGFR1, VEGFR1, VEGFR2, PDGFRα, and PDGFRβ by RON siRNA transfection was analyzed. Each column represents the mean ± SEM. * *p* < 0.05, compared with the siRNA control.

Moreover, TGFβ/Smad-dependent signals were reduced by RON-specific siRNA treatment in HK-2 and NRK49F cells, and phosphorylation of MAPK was reduced only in HK-2 cells. These results show that RON-specific siRNA treatment attenuates kidney fibrosis markers and their associated signal transduction. As shown in Figure 6, RON-specific siRNA treatment reduced the protein expression of RTKs such as IGFR, FGFR1, VEGF-R1, VEGF-R2, PDGFRα, and PDGFRβ in HK-2 and NRK49F cells, of which the degree was more prominent in NRK49F cells.

### 2.5. Effects of Src siRNA on EMT, Pro-Fibrotic Marker, and RTKs in HK-2 and NRK49F Cells

**Figure 7:**
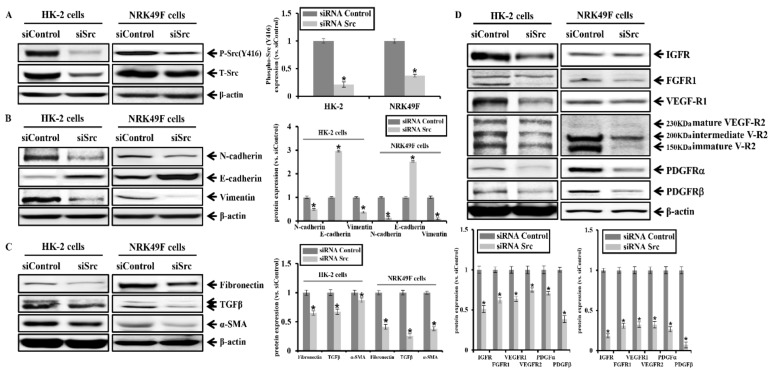
Effects of Src siRNA on EMT, pro-fibrotic marker, and RTKs in HK-2 and NRK49F cells. (**A**) Protein expression of the phosphorylated Src (Y416) and total Src by Src siRNA transfection was analyzed. (**B**) Protein expression of N-cadherin, E-cadherin, vimentin by Src siRNA transfection was analyzed. (**C**) Protein expression of fibronectin, TGFβ, and α-SMA by Src siRNA transfection was analyzed. (**D**) Protein expression of IGFR, FGFR1, VEGFR1, VEGFR2, PDGFRα, and PDGFRβ by Src siRNA transfection was analyzed. Each column represents the mean ± SEM. * *p* < 0.05, compared with the siRNA control.

We further examined whether knockdown of Src might be involved in the intracellular downstream signaling of RTKs. We examined the anti-fibrotic effects of Src by the transfection of Src siRNA in the HK-2 and NRK49F cells. Figure 7A shows the reduction of Src phosphorylation by Src-specific siRNA treatment. As shown in Figure 7B,C, knockdown by Src siRNA reduced the expression of EMT markers, N-cadherin and vimentin, and pro-fibrotic markers, fibronectin, TGFβ, and α-SMA. In addition, Src-specific siRNA treatment reduced the protein expression of RTKs such as IGFR, FGFR1, VEGF-R1, VEGF-R2, PDGFRα, and PDGFRβ (Figure 7D).

**Figure 8:**
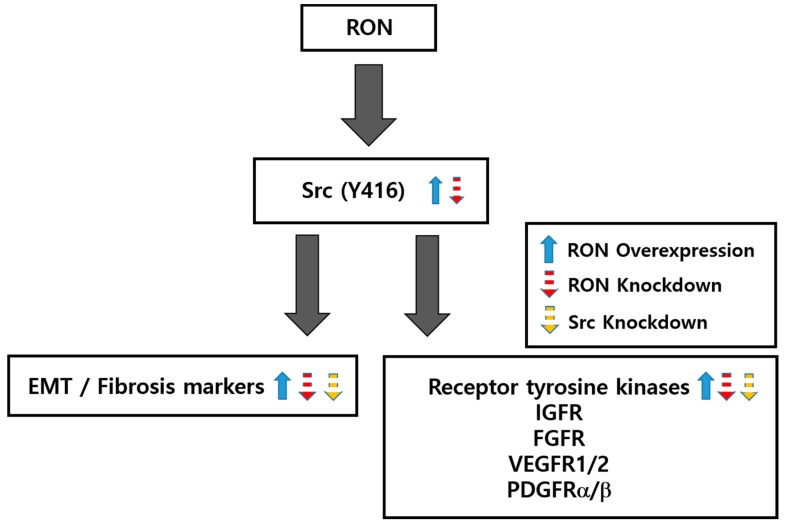
Schematic diagram of the regulation of EMT, fibrosis, and RTKs by RON and Src. Src phosphorylation, EMT, and fibrosis are regulated by RON overexpression and siRNA knockdown in HK-2 and NRK49F cells.

These results show that Src-specific siRNA treatment attenuates kidney fibrosis markers and their associated signal transduction and RTKs. Figure 8 is a schematic diagram illustrating the results of the above study.

## 3. Discussion

We demonstrated the regulation of EMT and fibrotic markers, as well as RTKs and Src phosphorylation, by RON overexpression and knockdown. The overexpression of RON induced EMT markers, such as N-cadherin and vimentin, and kidney pro-fibrotic markers, such as fibronectin, TGFβ, and α-SMA. In addition, the overexpression of RON increased the expression of RTKs in association with kidney fibrosis. In contrast, siRNA of RON reduced the protein expression of EMT and pro-fibrotic markers, and decreased the expression of RTKs. In addition, siRNA treatment of Src inhibited Src phosphorylation, reduced protein expression of EMT and pro-fibrotic markers, and reduced the expression of RTKs. These findings suggest that RON and Src regulates kidney fibrosis via the RON-Src signaling pathway (Figure 8).

Several studies have reported correlations between kidney diseases and RTK. FGFR1- and FGFR2- null mice cause early embryonic lethality [30,31], and gene inactivation of PDGFRβ in mice results in a lack of mesangial cells and pericytes of the glomerular capillaries [32]. In addition, decreased protein expression of IGF2R in the rat intrauterine growth restriction has been associated with decreased glomerular number, and IGFR has been associated with promoting nephrogenesis and kidney organogenesis [33]. These findings were associated with the release of various cytokines such as interleukin-1β (IL-1β), IL-6, IL-8, TNF-α, and monocyte chemoattractant protein-1, and multiple signaling [34,35].

We observed that various RTKs were controlled by RON. Two RTK monomers are generally dimerized upon ligand binding, and homodimer partners can be formed from the same protein molecule, whereas in some cases, different molecules from the same RTK family are heterodimerization to form a cell surface [36]. After dimerization, phosphorylation occurs at their cytoplasmic tyrosine residues, providing docking sites for other proteins, such as mitogen-activated protein kinases, Ras, PI3K/AKT, β-catenin, nuclear factor kappa B, and focal adhesion kinase, causing them to bind and activate their respective downstream signaling [20,37]. When RON is ligand dependent or independently dimerized, the α chain exists outside the cell, whereas the β chain containing tyrosine kinase regulatory elements crosses the cell membrane [38]. In the present study, RONα showed decreased protein level at 14 days of obstructed kidneys as compared to 7 days, and the expression patterns of RONβ and RON precursor forms increased at 14 days. It is speculated that extracellular RONα was separated from the cell membrane in severe degree kidney injury on UUO at 14 days; thus, only RON precursor and RONβ form showed increased protein expression.

Signal transduction of Src, TGFβ/Smad, STAT3, AKT, and MAPK is also activated in association with RTKs [39,40,41]. The present study also demonstrated that Src was regulated by the overexpression and knockdown of RON, and RTKs were reduced by the knockdown of Src. In addition, Smad signaling was activated by the overexpression of RON and reduced by RON knockdown. However, MAPK signaling was activated by the overexpression of RON and reduced by RON knockdown only in HK-2 cells, not in NRK49F cells. The regulatory mechanism by RON in NRK49F cells does not seem to be via MAPK signaling. Taken together, RON overexpression activates the increased production of fibrotic markers by Src/TGFβ/Smad signaling pathway.

Src kinase belongs to a family of non-receptor, intracellular protein tyrosine kinases. The Src family of protein kinases includes Src, Blk, Yes, Yrk, Fgr, Hck, Fyn, Lyn, and Lck, and six conserved domains include a N-terminal myristoylated segment, a SH2 domain, a SH3 domain, a linker region, a tyrosine kinase domain, and a C-terminal tail. Src family kinases interact with many cytoplasmic, nuclear, and membrane proteins to modify these proteins by phosphorylation of tyrosine residues [42,43], and interact with, and participate in, signaling from RTKs [44]. Activation of Src in the kidney has been reported to be associated with the pathogenesis of kidney tubular fibrosis, diabetic nephropathy, polycystic kidney disease, and obesity-induced kidney injury [45]. Src has been shown to mediate functional changes in the proximal tubule, as citrate reabsorption, that may have some effect on proximal tubule metabolism [46], and also phosphorylation of Src is increased in podocyte damage, such as focal segmental glomerulosclerosis [47]. As such, the role of Src in the current research has been shown to be important in kidney disease. Conversely, Src inhibition blocked kidney interstitial fibroblast activation and ameliorated kidney fibrosis [43]. In addition, Src is downstream of RON, and is involved in various signal transductions of phosphorylated RON. Phosphorylation of RON induced EMT by activating MAPK, GSK3β, and PI3/AKT signaling [26,48]. Our in vitro results also showed that siRNA inhibition of RON reduced the activation of Src pathways. In conclusion, these results suggest that RON regulates the expression of EMT and pro-fibrotic markers through the regulation of Src and Smad, and also has anti-fibrotic effects by regulating the expression of various RTKs.

## 4. Materials and Methods

### 4.1. Antibodies

The primary antibodies used were anti-rabbit antibodies against N-cadherin (40615), vimentin (5741), TGFβ (3711), IGF1R (3027), FGFR (3472), VEGFR1 (2893), PDGFRα (3164), PDGFRβ (3162), C-MET (8198), phosphorylated Smad 2/3 (8828), Smad 2/3 (3102), and Smad 4 (38454), all of which were obtained from Cell Signaling Technology, Inc. (Beverly, MA, USA). RONβ (sc-322), AQP-1 (sc-32737), ErbB4 (sc-8050), FLK-1 (VEGFR2, sc-393163), E-cadherin (610182), RONα (610744), fibronectin (610077), EGFR (MS-378-P1), smad6 (ab80049), and β–actin (a5316) were obtained from Santa Cruz Biotechnology, Inc. (Dallas, TX, USA), BD Biosciences (San Jose, CA, USA), Neomarkers (Fremont, CA, USA), Abcam (Alomone Laboratories, Ltd., Jerusalem, Israel), and Sigma-Aldrich Co. (St. Louis, MO, USA), respectively.

### 4.2. Animals

All methods were performed in accordance with the relevant guidelines and regulations. The experimental protocol was approved by the Animal Care Regulations (ACR) Committee of Chonnam National University Medical School (CNUH IACUC-18010, April 19, 2019). Male 8-week-old C57BL/6 J mice weighing 20 g were used for in vivo experiments. The mice were divided into three groups: Control (*n* = 6), unilateral ureteral obstruction for 7 days (UUO, *n* = 6), unilateral ureteral obstruction for 14 days (UUO, *n* = 6). In order to induce the obstructive nephropathy mice model, the operation was performed as follows. After anesthesia induction by using an intraperitoneal injection of ketamine (70 mg/kg), a midline incision was made to expose the abdominal cavity, and the left proximal ureter was ligated with 6-0 silk. Control mice were operated upon in the same way, except that no ligature was made. The mice had free access to standard chow and tap water. After 7 days and 14 days, the mice were sacrificed, and the left kidney was harvested for Western blot analysis or prepared for Immuno Fluorescence.

### 4.3. Cell Culture

Human kidney proximal tubular epithelial (HK-2) cells (ATCC, Manassas, VA, USA) and rat kidney interstitial fibroblasts (NRK49F) cells were cultured in Dulbecco’s modified Eagle’s Medium-F-12 medium (Sigma, St Louis, MO, USA) and Dulbecco’s modified Eagle’s Medium (DMEM) low glucose medium (WelGene, Daegu, Korea) supplemented with 10% (for DMEM-F12), 5% (for DMEM) fetal bovine serum, 100 U/mL penicillin, and 100 μg/mL streptomycin (Sigma) at 37 °C under a humidified 5% CO_2_ atmosphere. The cells were starved for one day with serum free media, and the cells were harvested at the end of treatment for further analysis.

### 4.4. Immunohistofluorescence (IHF)

Mouse kidney samples were fixed in 4% paraformaldehyde, embedded in paraffin, and cut into 2-μm-thick sections. Kidney sections were deparaffinized and rehydrated, blocked, and then washed with phosphate-buffered saline (PBS), and incubated with anti-RON and anti-AQP1 antibodies diluted in blocking buffer for 24 h at 4 °C. After washing, the sections were incubated with an Alexa Fluor 488 (green fluorescence) goat anti-rabbit antibody (Life Technologies, Carlsbad, CA, USA) or Cy3 goat anti-mouse diluted for 2 h. After washing, coverslips were mounted onto microslides using a ProLong Gold Antifade Reagent with DAPI (Life Technologies Corporation). Images were analyzed under the confocal laser-scanning microscope Leica TCS SP8 (Leica Microsystems, Mannheim, Germany).

### 4.5. Stable Cell Lines

HK-2 cells were transfected with 2 μg of empty vector (Mock) or RON DNA using 6 μL of Fusion HD reagent (Promega, Madison, WI, USA) in antibiotic-free DMEM-F12. Beginning at 1 day after transfection, transfectants were selected in DMEM-F12 containing 200 μg/mL zeocin, which was refreshed every 3 days for 2 weeks. Colonies surviving in the selection medium were collected and sequentially plated in 48-, 12-, and 6-well plates, and then 60- and 100-mm dishes. Cells stably overexpressing human RON were identified by immunoblotting with anti c-myc and anti β-actin antibodies or by PCR analysis using zeocin primers 5′-ATGGCCAAGTTGACCAGTGCCGTT-3′ (forward) and 5′-GTCCTGGTCCTCGGCCACGAAGTG-3′ (reverse) [49]. A loading control was analyzed by using GAPDH primers 5′-ACCACAGTCCATGCCATCAC-3′ (forward) and 5′-TCCACCACCCTGTTGCTGT-3′ (reverse).

### 4.6. Transient Transfection of Plasmid Construct, RON

PCMV6-Entry RON (cat. RC212786) was purchased from Origene (Rockville, MD, USA). The human RON was subcloned into EcorI/PmeI site of pcDNA4 vector. PcDNA4-RON was introduced to NRK49F cells by FuGene HD reagent (Promega). Two days after transfection, we identified the overexpression of RON in NRK49F cells by Western blot analysis.

### 4.7. siRNA Knockdown

RNA interference of RON and Src was performed using an MST1R human siRNA oligo duplex from Origene (Rockville, MD, USA. Cat. SR302976) and on-target plus smartpool human Src from Dhamacon ((Lafayette, CO, USA. Cat. L-003175-00). Briefly, cells were transfected with the indicated concentration of siRNA (30 nM and 50 nM) using DhamaFECT 1 transfection reagent according to the manufacturer’s protocol. Cells transfected with control siRNA (Santa Cruz, Cat. sc-37007) were used as controls for direct comparison.

### 4.8. Western Blot Analysis

The cells were harvested, washed twice with ice-cold phosphate-buffered saline (PBS), resuspended in lysis buffer, and sonicated briefly. After centrifugation, the supernatants were prepared as protein extracts, and the protein concentrations were measured using a Pierce^®^ BCA Protein Assay Kit (Pierce Biotechnology, Inc., Rockford, IL, USA). Equal concentrations of protein were separated on 12% sodium dodecyl sulfate polyacrylamide gels, and the proteins were transferred onto nitrocellulose membranes. The blots were blocked with 5% milk in PBS-T for 1 h. The blots were then incubated overnight at 4 °C with the primary antibodies, which was followed by incubation with the anti-rabbit horseradish peroxidase-conjugated antibodies, as described previously [50]. The labeling was visualized using an enhanced chemiluminescence system.

### 4.9. Statistical Analysis

Results are expressed as mean ± SEM. Multiple comparisons among the groups were made by one-way ANOVA and post hoc Tukey HSD test. In this study, *p* values < 0.05 were considered significant.
